# An exploration of contextual dimensions impacting goals of care conversations in postgraduate medical education

**DOI:** 10.1186/s12904-016-0107-6

**Published:** 2016-03-21

**Authors:** Amanda L Roze des Ordons, Jocelyn Lockyer, Michael Hartwick, Aimee Sarti, Rola Ajjawi

**Affiliations:** Department of Critical Care Medicine and Division of Palliative Medicine, South Health Campus Intensive Care Unit, University of Calgary, 4448 Front St SE, Calgary, AB T3M 1M4 Canada; Cumming School of Medicine, University of Calgary, Calgary, AB Canada; Divisions of Critical Care and Palliative Care, University of Ottawa, Ottawa, ON Canada; Divisions of Critical Care and Palliative Care, University of Calgary, Ottawa, ON Canada; Centre for Research in Assessment and Digital Learning, Deakin University, Melbourne, VIC Australia

**Keywords:** Communication, Goals of care, Context, Postgraduate medical education, Qualitative

## Abstract

**Background:**

Postgraduate medical trainees are not well prepared difficult conversations about goals of care with patients and families in the acute care clinical setting. While contextual nuances within the workplace can impact communication, research to date has largely focused on individual communication skills. Our objective was to explore contextual factors that influence conversations between trainees and patients/families about goals of care in the acute care setting.

**Methods:**

We conducted an exploratory qualitative study involving five focus groups with Internal Medicine trainees (*n* = 20) and a series of interviews with clinical faculty (*n* = 11) within a single Canadian centre. Thematic framework analysis was applied to categorize the data and identify themes and subthemes.

**Results:**

Challenges and factors enabling goals of care conversations emerged within individual, interpersonal and system dimensions. Challenges included inadequate preparation for these conversations, disconnection between trainees, faculty and patients, policies around documentation, the structure of postgraduate medical education, and resource limitations; these challenges led to missed opportunities, uncertainty and emotional distress. Enabling factors were awareness of the importance of goals of care conversations, support in these discussions, collaboration with colleagues, and educational initiatives enabling skill development; these factors have resulted in learning, appreciation, and an established foundation for future educational initiatives.

**Conclusions:**

Contextual factors impact how postgraduate medical trainees communicate with patients/families about goals of care. Attention to individual, interpersonal and system-related factors will be important in designing educational programs that help trainees develop the capacities needed for challenging conversations.

**Electronic supplementary material:**

The online version of this article (doi:10.1186/s12904-016-0107-6) contains supplementary material, which is available to authorized users.

## Background

Communication and decision-making about care near the end of life (EOL) is difficult for patients, their families, and healthcare providers. Goals of care conversations in the acute care setting are often laden with the emotional stress of difficult decisions, absence of a previous relationship, and uncertainties around diagnosis and prognosis [1]. The consequences have been well described, including misinterpretation of patient preferences [2, 3], emotional distress amongst trainees [3, 4], and adverse psychological outcomes amongst patients and families [5].

How physicians communicate with patients and families about palliative and EOL care varies across settings [6] and impacts the illness experience [7] and treatment decisions [8]. A number of contextual factors appear to impact EOL communication and decision-making in the clinical environment. These factors operate at the individual, interpersonal and systems levels, and include self-efficacy and patient expectations, role models and mentors, and the specialty of clinical practice and time constraints, respectively [9, 10]. Furthermore, these factors interact and become entwined, leading educators and administrators to recognize the complexity of improving patient care through attempts to modulate clinician behavior; previous research has mostly focused on individual factors and most practice-level interventions have shown only limited or modest success [11]. Expanding our perspectives beyond the individual may provide important insights about how to optimize EOL communication and decision-making.

Complexity theory has been used to make sense of interactions between contextual factors and guide the design and implementation of interventions to enhance patient care. Complexity theory provides a framework to examine multiple interactions within a context of care [11], looking beyond single cause-effect mechanisms [12]. Context has a critical influence on outcomes of educational interventions, impacting whether the intervention is effective, for which learners, under which circumstances, and why [13]. In the acute care setting, trainees, their preceptors, other healthcare providers, and patients and families interact with one another and with sociocultural and organizational factors in the healthcare and education systems. These interacting contextual factors convey and create implicit and explicit messages that shape how care is delivered and how trainees learn.

Despite education, trainees struggle with conversations and decision-making related to palliative and EOL care [4]. While these difficulties have been attributed to trainee factors, information exchange, and limitations of teaching and assessment, the impact of the clinical environment has been relatively unexplored. Examining the clinical environment and how it impacts goals of care discussions may help us better understand why these discussions are difficult and how they might be facilitated.

The purpose of this study was to explore contextual factors that influence conversations between trainees and patients and their families about goals of care, to inform future educational interventions within the clinical setting. We were particularly interested in factors that were operational at the individual, interpersonal, and system levels, and the consequential impact of these factors on trainees. While the study focused on palliative and EOL care, it is likely that similar factors may impact on goals of care discussion involving patients with other serious illnesses where the course is uncertain.

## Methods

We undertook a qualitative study of the context for communication skills teaching and learning in postgraduate medical education (PGME). The study was conducted within an Internal Medicine (IM) training program at the University of Ottawa. The program is 3 years in duration; each clinical rotation is 4 weeks, and at least 7 rotations per year are hospital-based. The formal curriculum includes 2–6 h per year of communication skills teaching, involving a combination of didactic material, role play and objective structured clinical examinations; first year trainees also participate in simulated practice. The communication skills training is guided by the Royal College of Physicians and Surgeons of Canada (RCPSC) CanMEDS Objectives of Training for Internal Medicine [14]. Patient care is provided by an interprofessional team, and goals of care conversations are the responsibility of attending physicians and medical trainees. Attending internal medicine physicians and trainees review all patients daily and attending physicians may attend clinic in the afternoon. Trainees provide a large portion of patient care during the day and at night, with attending physicians readily available as needed.

### Data sources

We obtained data from focus groups and interviews held between May and August 2013. All Internal Medicine (IM) trainees in postgraduate years (PGY) 1 to 3 were invited to participate in focus groups. For interviews, clinical faculty from the Department of Medicine were identified through purposive sampling [15]. Focus groups were led by an experienced qualitative research assistant (NO) and the primary investigator (AR); interviews were conducted by the primary investigator (AR). Focus groups and interviews explored trainee experiences in communicating with patients and families, and clinical faculty experiences with communication teaching in the clinical setting (Additional file 1). All participants provided written informed consent.

### Data analysis

Focus groups and interviews were audio recorded and transcribed verbatim by a professional transcriptionist. Data were analyzed using thematic framework analysis [16], beginning with listening to audio recordings and multiple close readings of each transcript. Two investigators (AR, RA) independently analyzed five transcripts (1 focus group and 4 interview transcripts) to inductively develop a preliminary coding framework. The coders discussed and negotiated the coding framework to establish consensus. AR analyzed and indexed all remaining transcripts using constant comparison analysis [15]. RA examined the quotes assigned to each code for consistency of application. The data were then collapsed into themes and subthemes, and themes mapped to individual, interpersonal, and system dimensions, examining the data that focused on the contextual factors influencing goals of care conversations. The project received approval from the University of Ottawa Research Ethics Board.

## Results

We conducted 5 focus groups with 20 of 93 (21.5 %) IM trainees (PGY 1, *n* = 12; PGY 2, *n* = 5; PGY 3, *n* = 3), and 11 interviews with clinical faculty from the Department of Medicine. Each focus group and interview was 40–60 minutes in duration.

**Fig. 1:**
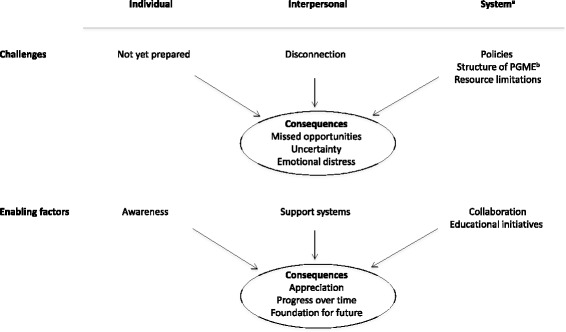
Individual, interpersonal and system-related challenges and enabling factors impacting goals of care discussions and the consequential impact on postgraduate trainees. Legend: **a** Healthcare system and Education system. **b** Postgraduate medical education

Qualitative data were categorized as individual, interpersonal and system-related dimensions that impact goals of care communication, and consequences of these contextual dimensions. Themes and subthemes within each dimension clustered under challenges and enabling factors, as illustrated in Fig. 1.

### I. Individual dimensions

Individual dimensions were those specific to an individual trainee or faculty member, including personal characteristics and experiences, knowledge, attitudes, and behaviours. Two major themes impacting goals of care conversations in relation to the individual were *Not yet prepared* as a challenge, and *Awareness* as an enabling factor.

#### Challenge—Not yet prepared

Trainees identified that discussions were impeded by a societal culture focused on cure, their own discomfort with death and dying, infrequent teaching around goals of care, and uncertainty in approaching goals of care conversations.

*The issues around death is really something that I don’t feel comfortable doing. (Trainee 1, Focus Group [FG] 1)*

*You really don’t get any formal or informal observation or I haven’t. You talked about one of your staff coming in with you to break bad news… I've never had that. (Trainee 5, FG1)*

Inexperienced trainees assumed responsibility for goals of care conversations and perceived patients and families as unprepared to discuss palliative and EOL planning, regardless of acuity or severity of illness.

*We are the ones having these conversations in the middle of the night and we’re not really the best people to… say what the prognosis is or will this really help because we don’t necessarily know. (Trainee 14, FG4)*

Trainees also held high self-expectations to be “good communicators” and were critical of their capacity to discuss goals of care; experience did not always translate into self-confidence.

*I'm more of an intro-reflective person. I leave the room and I'm like, “Oh, you're an idiot. You did this wrong, this wrong.” (Trainee 5, FG1)*

*Being able to identify the type of person you're talking to and being able to deliver information in different ways will be an ongoing challenge. (Trainee 19, FG5)*

Some clinical faculty felt inexperienced in teaching about goals of care conversations and in providing learner-centered feedback; they attributed these anxieties to the absence of direct supervision and feedback on goals of care conversations during their own training and faculty development. The resultant lack of confidence and reluctance to provide critical feedback contributed to challenges integrating teaching into clinical practice.

*Maybe some staff themselves weren’t given formal education around communication and maybe they don’t feel skilled enough to give us feedback. (Trainee 9, FG2)*

*I find a better way to evaluate them is to try and say some good things, even if you think they’re terrible because, you know, you can’t just give someone bad news. (Clinical faculty, Interview [i] 4)*

#### Enabling factor—awareness

Trainees described empathy toward patients and families, and were aware of the importance of discussing goals of care.

*Training [about] how to approach code status because that’s a discussion that we do often and I don’t think we do it well. (Trainee 8, FG2)*

Clinical faculty spoke of the need to incorporate goals of care conversations into clinical curricula and were interested in developing their skills in teaching and providing feedback about discussing goals of care.

### II. Interpersonal dimensions

Interpersonal dimensions were factors co-created through interaction between healthcare providers, trainees, and patients and their families. Two major themes influencing goals of care conversations that reflect interpersonal dimensions were *Disconnection* as a challenge and *Support systems* as an enabling factor.

#### Challenge—disconnection

A hierarchical relationship between trainees and clinical faculty and independence in communicating about goals of care was evident in the clinical setting. Clinical faculty assigned trainees responsibility for discussing goals of care but described not always feeling confident in trainees’ ability to have these discussions. Trainees and clinical faculty described learning through clinical experience, rooted in a precedence of training that often uncouples clinical practice from supervision or feedback.

*Yeah, a lot of our learning has been trial by error… fumbling your way. (Trainee 2, FG1)*

*I remember many instances as a resident, just talking about like “Do you want the breathing tube?” But nobody listened to me have that conversation ever. Nobody told me how to approach it. (Clinical faculty, i2)*

*Staff rounds in the morning, clinic in the afternoon. A lot of family meetings on the other hand take place in the afternoon, so the staff are not there. It’s hard to squeeze it all into three hours in the morning. (Clinical faculty i7)*

Trainees, in turn, expected patient autonomy in decision-making around goals of care, while at the same time recognizing that physicians may frame conversations to achieve the decision they believe most medically appropriate. Trainees recognized that cognitive biases and physician-driven agendas were contradictory to the emphasis on patient autonomy and shared clinical decision-making taught in the classroom.

Absence of a prior relationship with patients created additional challenges in achieving a shared understanding about the plan of care.

*Sometimes you need [code] status when you’re just seeing the person for the first time and the family’s not really aware of how severe their current issue is and they kind of push for everything, and you don’t really know this person yourself. Even though you know that CPR and ICU [are] unlikely to help. (Trainee 7, FG1)*

Trainees and clinical faculty also expressed discomfort in acknowledging their own and patients’ emotions, focusing on information over the emotional aspects of illness.

*The patient had been very stoic but the family just like broke down and was crying. I wasn’t expecting this. (Trainee 10, FG2)*

*Some staff can't deal with the social stuff or the more emotional stuff but are excellent from like a medical expert point of view. The feelings part, they're not so great at. (Trainee 13, FG3)*

#### Enabling factor—support systems

Clinical faculty support for trainees was available when sought, and peers were a source of moral support. Some clinical faculty deliberately role modeled and directly observed trainees discussing goals of care, making the effort to teach while facilitating optimal patient care.

*I tried to answer the [family’s] questions as much as possible but in the end, I went out and talked to the staff and they were like, ‘Let’s talk to them again’, and it ended up happening the next day that things were clarified. (Trainee 8, FG2)*

*I try not to be the primary communicator with the patient. I like the resident to do that, and I observe. (Clinical faculty, i3)*

### III. System dimensions

System dimensions encompassed themes reflecting healthcare and education system influences on goals of care conversations with *Policies,* the *Structure of medical education* and *Resource limitations* as challenges, and *Collaboration* and *Educational initiatives* as enabling factors.

#### Challenge—policies

Policies within the healthcare system that expected goals of care designation at hospital admission generated significant uncertainty and distress amongst trainees, patients and families.

*Two in the morning you're having a consult in Emergency with a family you have no rapport with at all… and you're trying to find out what they want ‘cause you have to fill out this sheet and you don’t know what to do. (Trainee 15, FG4)*

Forms for documenting goals of care were the focal point of conversations and decision-making. Trainees also noted that an emphasis on patient flow and numbers was often prioritized over patient care and de-emphasized the importance of communication.

#### Challenge—structure of postgraduate medical education

The structure of PGME, with short rotations, work-hour restrictions, and classroom-based teaching, generated suboptimal conditions for mentoring, role modeling, and assessing trainee communication with patients over time.

*It’s often hard to do the evaluation because you may get one week with them… I’m always getting evaluations on people I can hardly recognize; it becomes a bit artificial. (Clinical faculty, i4)*

The structure of residency education also reduced the number of opportunities to establish rapport and have meaningful conversations with patients. Frequent handovers and inconsistent patient assignments led to diffusion of responsibility for goals of care conversations and decision-making.

*And then you're post call and you hope that somebody else picked up [the conversation] but you don’t really get to see that progression the next day, and then somebody else gets assigned the patient. (Trainee 15, FG4)*

Despite identified objectives, education about goals of care conversations was inconsistently incorporated into the formal and informal curriculum. The medical expert role was prioritized and communication conceptualized as a “touchy-feely” and “soft skill” by trainees and clinical faculty. Communication teaching that did occur was most often didactic rather than skills-based, with infrequent role modeling, direct observation or feedback.

Competition for fellowship positions further impaired feedback on communication. Trainees perceived feedback as helpful when offered, although hesitated to solicit feedback that could lead to unfavorable evaluations.

*There's so much pressure to get good evaluations and it's so competitive that you don't want to give anyone the chance to say anything negative, even if it means improving your training. (Trainee 13, FG3)*

#### Challenge—resource limitations

Time constraints generated by low physician to patient and clinical faculty to trainee ratios created challenges for discussing goals of care and for observing and providing feedback to trainees on these conversations, respectively.

*It’s difficult when you are seeing five patients and you’re admitting all of them and you want to have a [goals of care] discussion. (Trainee 6, FG1)*

*If you have a very busy month as staff, and you've got few residents and sick patients, it's hard to do the ideal job of watching every resident several times do different meetings; it's just impossible. (Clinical faculty, i6)*

Trainees also described insufficient quiet space for such conversations, and limited availability of translators and social work support outside of weekday hours.

#### Enabling factor—collaboration

Trainees and faculty recognized the strengths of interprofessional collaboration, with palliative care consultants, nurses, and social workers bringing experience in discussing goals of care and access to resources.

*[A family meeting] where one staff and one social worker and a nurse was there… And I think that’s important, what you’re saying when you bring everyone together, and everyone in the team are all on the same page and you can carry that forward. [Trainee 16, FG4]*

#### Enabling factor—educational initiatives

Trainees perceived that basic communication skills learned in medical school facilitated goals of care conversations. Trainees also appreciated educational initiatives that were gradually being introduced into postgraduate training, including group discussions and simulation.

*At the sim[ulation] centre they actually gave you a reasonable framework, I’m still using that framework. (Trainee 7, FG1)*

In addition to being an instrument of research, the focus groups also promoted self-reflection and sharing of experiences, through which participants gained insights and received support from peers. Finally, regulatory bodies were recognized as supportive of initiatives to integrate communication teaching and assessment into PGME.

### IV. Consequences of interacting individual, interpersonal and system factors

Challenges and enabling factors within the individual, interpersonal and system dimensions affected trainee education and their experience of discussing goals of care. Contextual challenges resulted in missed opportunities, uncertainty, and emotional and moral distress.

The following quote illustrates how interpersonal and system factors generate difficulties in establishing and sustaining rapport and trust between trainees, their peers, and patients. The emotional toll on trainees is evident in the phrases ‘the whole thing just fell apart’ and ‘robbed of dying’.

*These short rotations and post-call; I’d like to think that I'm establishing rapport but… somebody else takes your patient and that whole thing just falls apart. And people get robbed of dying because all it took was one statement from one person one time and the rapport that you have [had] so little chance to build is gone. (Trainee 16, FG4)*

Trainees were uncertain about the content and process of goals of care conversations, paralleled by a lack of confidence amongst clinical faculty in teaching and providing feedback on these conversations, and system factors driven by technical tasks, such as completing forms. This contributed to trainee insecurity and cynicism, and feelings of abandonment, disappointment, and frustration about their education and clinical experiences in discussing goals of care with patients and families.

*I just felt really stuck, just really alone and isolated in this conversation that you basically have to have because you’ve got to fill out all the sheets. (Trainee 15, FG4)*

*It’s never about the communication; it’s about getting the work done. And I think that the shift really needs to change in our philosophy before we become robots. (Trainee 1, FG1)*

While some clinical faculty expressed cynicism, others spoke of attachment to their patients. Moral distress was evident in stories recounted about experiences of goals of care conversations and the resulting decisions.

*Some of the care that I've given elderly patients in ICU and on the wards when they’ve coded has just horrified me, it's almost like torturing someone as they die. And, you know, that’s shaken me. (Clinical faculty, i7)*

The enabling factors have resulted in learning, appreciation, and an established foundation for future educational initiatives around goals of care conversations.

*And the staff actually briefed me on how to approach it. I had a fabulous [preceptor] at this time. And he walked me through what my plan should be and how I should go about it. And we went into the room together and he asked me to take charge of the conversation, so I did. (Trainee 4, FG1)*

Clinical faculty commented on improvements in trainees’ capacity to discuss goals of care as they progressed in their training, and trainees appreciated the teaching and support they had received. Finally, current educational interventions have generated the awareness, enthusiasm and skilled educators required for successful development and implementation of future initiatives.

## Discussion

Through focus groups and interviews, we have identified a number of contextual challenges and factors that enable goals of care conversations in the acute care setting. Previous research on palliative and EOL communication in PGME has largely focused on needs [17, 18], what to teach and how [19], and more recently on associated outcomes [20]. The current study is unique in characterizing contextual aspects that may impact trainee conversations with patients and families about goals of care, providing direction for contextualized learning initiatives at individual, interpersonal and systems levels.

At the individual level, goals of care conversations require acknowledgement of a future when life-prolonging therapies are no longer of benefit [21] and experience in facilitating such discussions and decision-making [22, 23]. Trainees in our study felt unprepared, yet were aware of the importance of conversations around goals of care and the central role of the therapeutic relationship in facilitating difficult discussions. This confirms findings from other studies that have shown current medical training inadequately prepares trainees for communication about palliative and EOL care [18], with consequential impact on patients, families [23], and healthcare providers [3, 4]. Nonetheless, the resultant cognitive dissonance has established the motivational prerequisite for effective learning.

It is recognized that ‘No dialogue takes place in a vacuum; there is a whole world surrounding it’ [24]. This world of interpersonal and system-related factors become intertwined. At the interpersonal level, goals of care conversations require rapport and trust, and the resources of time and space [22, 23]. The therapeutic relationship between patients, families and physicians is foundational for difficult conversations; addressing goals of care early on in hospitalization, when such a relationship has not yet been established, may undermine trust and contribute to iatrogenic suffering [25]. With communication bound to self-concept [9], the analogous professional relationship between trainees and clinical faculty is essential for role modeling and provision of developmental feedback that feels safe to the learner and can be heard [26]. Impoverished relationships created by a hierarchy between trainees and clinical faculty and a focus on independence interferes with the learning process [27]; autonomy may be disrespectful when undesired or assigned prematurely and thus experienced as abandonment. Responsibility contributes to development of competence, and graded supervision until competence has been demonstrated is equally applicable to communication as procedural skills [28]. While faculty and interprofessional colleagues were available to support trainees in our study, the hesitance to request feedback and support may have been partly related to the dominant culture of independent learning [9, 28].

At the systems level, the structure of PGME, policies around goals of care documentation, and resource limitations further impair learning to discuss goals of care and interfere with opportunities for these conversations. The impact of frequent transitions on trainees, faculty, and patients has been previously described [29, 30]; fleeting encounters over enduring relationships, and unprofessional behaviours adopted to cope with an ever-changing context diminish trainees’ capacities for humanistic and meaningful conversations with patients about palliative and EOL care. The optimal timing of goals of care conversations in hospital remains controversial [31]. Time of day, physical space, healthcare provider workload, experience of available staff, acuity and severity of patient condition, and availability of surrogate decision makers are only some of the factors that may impact the best time to discuss goals of care. Earlier conversations under suboptimal conditions may lead to misrepresentative decisions, yet delaying conversations until conditions are optimized risks treatments inconsistent with patient values and patient deterioration prior to decisions having been made [32, 33]. Furthermore, time pressures and policies around documentation may generate a task-focused mindset. Forms for designating goals of care may be misconstrued as a guide for communication and decision-making rather than being recognized as a means of conveying to other healthcare providers the outcome of a broader conversation about a patient’s goals and values in the context of their current medical condition.

This study has several implications for teaching communication skills related to palliative and other patient care situations where goals of care need to be explicitly negotiated. Modulating each contextual factor described would be a daunting task. Adopting a complexity theory lens, the interconnectedness between individual, interpersonal and systems can be appreciated and more holistic approaches adopted accordingly. For example, a relationship-centered approach to patient care and medical education, underpinned by humanistic values, mutual respect, shared decision-making and reciprocal influence, would value learning through sharing knowledge and experiences [26, 34]. Relational learning may help trainees learn from and adapt to challenges within a complex and unpredictable environment, fostering the capacity to transform as the world around them changes. Applied to communication about goals of care, such an approach could involve contextualized learning in the clinical setting through patient narratives, role modeling and guided reflection, feedback from multidisciplinary preceptors as well as patients and families, and mentorship from peers and faculty [35]. Elements of the clinical workplace could also be integrated into the classroom, through simulation scenarios derived from challenging conversations, and team-based educational initiatives. Faculty development courses to develop skills in providing feedback to trainees on communication in challenging conversations may also be helpful.

Our research has generated a number of questions for further study. For example, as interpersonal and systems levels factors are identified, it would be helpful to determine which could be modulated to enhance palliative and EOL care conversations and subsequent patient care. Are there optimal ways to teach skillful communication? Finally, further research is needed to learn how individual, interpersonal, and systems level challenges and enabling factors impact communication in contexts beyond palliative care, particularly in other areas of medicine where outcomes are uncertain.

There are limitations to the current study. The current research involved a single postgraduate training program based at several urban teaching hospitals. Reporting the context and participant characteristics allows readers to assess for transferability beyond the setting of this study [15]. Participation was voluntary, introducing potential self-selection bias; participants may therefore have represented a subgroup with a particular interest in communication such that results may not reflect those of the larger group. Patient, family, and non-physician healthcare provider perspectives were not elicited in the study; such perspectives would add to our understanding of the context for goals of care conversations, a consideration for future research. Also, medical professionals have a tendency to be self-critical and external perspectives may have shown appreciation of the efforts taken to engage in challenging conversations. While the investigators’ work in critical care and palliative medicine has influenced the study design, results, and interpretations, these interconnections allow for awareness of contextual dynamics.

## Conclusions

The current study has identified and characterized individual, interpersonal and system-related factors influencing postgraduate trainee conversations with patients and families about goals of care within the acute care setting, and the impact of these contextual factors on trainees. While the challenges currently overshadow the enabling factors, the latter have established a foundation upon which further education about communication in palliative and other challenging clinical contexts can be developed. Characterizing and addressing the context in which these future educational interventions are embedded will be important for their success. Applying complexity theory through a lens of relationship-centered care may foster the culture of learning and support needed for effective education and subsequent conversations and decision-making around goals of care.

## Ethics approval and consent to participate

Ethics approval for this study was obtained from the University of Ottawa Research Ethics Board. All participants provided written informed consent.

## Additional file

Additional file 1: Focus group and interview guides. (DOCX 104 kb)

## Abbreviations

EOL: end of life
IM: internal medicine
PGME: postgraduate medical education
RCPSC: Royal College of Physicians and Surgeons of Canada
