# Supplementary material for: An exploration of contextual dimensions impacting goals of care conversations in postgraduate medical education
Source: BMC Palliat Care. 2016 Mar 21;15:34. doi: 10.1186/s12904-016-0107-6 (PMC4802849; doi:10.1186/s12904-016-0107-6)
Supplement: Additional file 1: — Focus group and interview guides. (DOCX 104 kb) [file 12904_2016_107_MOESM1_ESM.docx]

**Additional file 1 – Focus Group and Interview Guides**

**I. Trainee Focus Group Questions:**

1. What aspects of communicating with patients and their families do you find to be especially challenging?
   1. What is it about these situations that is challenging?
      1. Probes
         - Who meets with patients and their families? Whose responsibility is this?
         - Can you recall a patient with whom you had to communicate regarding disclosing bad news, discussing a care plan or ‘code status’? Or have observed? Who meets with patients and families on the wards (staff, residents, students?)
         - Please describe the encounter. What was done well? What could have been done better? Were you alone or with another resident or staff? Did you feel supported? Did you feel comfortable? Was there any teaching or debriefing around the encounter? Do you feel that you are confident in these situations?
         - Have you ever found yourself in a situation where you did not feel comfortable communicating with a patient or family? What did you do?
         - Is it typically easy or difficult to communicate with patients and families?
2. Are you satisfied with the formal communication teaching you have received during your residency?
   1. Why/why not?
      1. Probes – What are the strengths of the program? What are the weaknesses?
   2. If you were program director, how would you change these aspects of your training?
   3. What barriers exist to making these changes?
      1. Probes – Are there any competing tasks or constraints? Eg: time, etc.
3. What has been your experience with informal learning of communication during your clinical rotations?
   1. Individual factors
      1. Probes – Attitudes, confidence, fears, personal attachment, training, preparation
   2. System factors
      1. Probes – Any time constraints, preceptor assumptions/expectations, preceptor role modeling, preceptor observation/feedback, hospital vs clinic environment)
4. What strategies would you find to be helpful for a) learning, b) developing, and c) maintaining skillful communication?
   1. Why do you think these strategies would be effective for you?
      1. Probes – Have you found these strategies to be effective in other situations?
5. When should communication be formally taught?
   1. Probes – Communication does not need to be taught, medical school (pre-clerkship and/or clerkship), residency (PGY1 vs 2 vs 3 vs 4 vs 5 vs all PGY years), continuing professional development, all of the above

**II. Clinical Faculty Interview Questions:**

1. When should communication be formally taught?
   1. Are there limitations of formal teaching?
2. Which aspects/areas of communication do you find to be most challenging for residents?
   1. What is it about this that is particularly challenging for residents?
3. Is communication a topic that you address during clinical teaching on the hospital wards? In clinic?
   1. If not, why is that? What are some barriers/challenges that exist? How could these be overcome?
   2. If yes, which methods do you find to be effective?
   3. What is your level of comfort in teaching communication to residents? Do you think your colleagues feel the same way?
   4. Have you previously received training in communication?
   5. Would you be interested in participating in train the trainer sessions?
4. What are the main challenges/barriers to improving residents’ communication?
   1. What would be some challenges to implementing a structured communication training program?
   2. How could those challenges be overcome?
